# Y6 Organic Thin‐Film Transistors with Electron Mobilities of 2.4 cm^2^ V^−1^ s^−1^ via Microstructural Tuning

**DOI:** 10.1002/advs.202104977

**Published:** 2021-12-02

**Authors:** Edgar Gutierrez‐Fernandez, Alberto D. Scaccabarozzi, Aniruddha Basu, Eduardo Solano, Thomas D. Anthopoulos, Jaime Martín

**Affiliations:** ^1^ POLYMAT University of the Basque Country UPV/EHU Av. de Tolosa 72 San Sebastián 20018 Spain; ^2^ King Abdullah University of Science and Technology (KAUST) KAUST Solar Center (KSC) Thuwal 23955 Saudi Arabia; ^3^ ALBA Synchrotron Light Source NCD‐SWEET Beamline Cerdanyola del Vallès 08290 Spain; ^4^ Ikerbasque Basque Foundation for Science Bilbao 48013 Spain; ^5^ University of A Coruña Group of Polymers Centro de Investigacións Tecnolóxicas (CIT) Esteiro Campus Ferrol 15471 Spain

**Keywords:** electron mobility, nonfullerene acceptors, organic thin‐film transistors, polymorphism

## Abstract

There is a growing demand to attain organic materials with high electron mobility, *μ*_e_, as current reliable reported values are significantly lower than those exhibited by their hole mobility counterparts. Here, it is shown that a well‐known nonfullerene‐acceptor commonly used in organic solar cells, that is, BTP‐4F (aka Y6), enables solution‐processed organic thin‐film transistors (OTFT) with a *μ*_e_ as high as 2.4 cm^2^ V^−1^ s^−1^. This value is comparable to those of state‐of‐the‐art n‐type OTFTs, opening up a plethora of new possibilities for this class of materials in the field of organic electronics. Such efficient charge transport is linked to a readily achievable highly ordered crystalline phase, whose peculiar structural properties are thoroughly discussed. This work proves that structurally ordered nonfullerene acceptors can exhibit intrinsically high mobility and introduces a new approach in the quest of high *μ*_e_ organic materials, as well as new guidelines for future materials design.

## Graphical abstract

n‐channel organic thin‐film transistors exhibiting electron mobility values as high as 2.4 cm^2^ V^−1^ s^−1^ are produced with the benchmark nonfullerene acceptor used in organic solar cell, that is, BTP‐4F (aka Y6). These properties stem from crystallization of Y6 in a crystalline structure featuring molecularly flat, highly ordered, and textured domains.

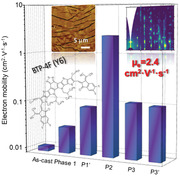

## 1 Introduction

The recent years have witnessed a rapid improvement in the performance of organic solar cells (OSCs) due mainly to the use of increasingly optimized fused‐ring electron‐accepting compounds, that is, the so‐called nonfullerene acceptors, NFAs. The main strength of NFAs, compared to their fullerene‐based counterparts, is considered to be a strong absorption in the solar spectrum that is complementary to the absorption region of common donor materials.^[^ ^1^, ^2^ ^]^ Less attention has been paid, however, to their charge transport efficiency.

Charge transport in molecular semiconductors is known to be closely connected to their molecular packing motif in the solid‐state and their crystalline quality. Indeed, the former determines the transfer integrals between neighboring molecules, while the latter allows the reduction of static disorder and hence the achievement of low trap density.^[^ ^3^ ^]^ Structural analysis of most efficient NFAs has revealed the formation of mesh‐like packing motifs in the solid‐state, promoting multiple charge percolation pathways and efficient electron transfer in multiple directions.^[^ ^4^ ^]^ For example, the benchmark NFA, BTP‐4F (also known as Y6),^[^ ^5^ ^]^ has been proposed to pack into a crystalline lattice that includes two distinctive sets of transporting channels.^[^ ^6^ ^]^ This structure is thus expected to play an important role in the high electron mobility, *μ*_e_, and current density measured in Y6‐containing solar cells.^[^ ^4h^ ^]^ Indeed, the intrinsically good transport properties of Y6 crystals have been demonstrated in single‐crystal organic thin‐film transistors (OTFTs) that displayed an *μ*_e_ of 1.9 cm^2^ V^–1^ s^–1^.^[^ ^7^ ^]^ This remarkable value suggests a great potential of structurally ordered Y6—and likely other NFAs, not only in the photovoltaic arena but also in further applications that require high *μ*_e_.

In this paper, we investigate the solid‐state structure of Y6 thin films and its correlation with charge transport. Our study reveals a rich polymorphism, including five polymorphs, in addition to an oriented glassy microstructure; and provides the processing routes toward all of them. The most important finding from this study is that a readily achievable phase, the so‐called phase 2, enables solution‐processed OTFTs with a *μ*_e_ as high as 2.4 cm^2^ V^−1^ s^−1^, that is, a value comparable to those of the state of the art thin film n‐channel OTFTs.^[^ ^8^ ^]^ We discuss the implications of structural characteristics of phase 2 for such efficient electron transport and rationalize the origin of this microstructure.

## 2 Results and Discussion

We begin our discussion by reporting the different polymorphic phases and microstructures that we identified in solution‐processed (spin cast) Y6 films. Unless otherwise stated, the analyzed samples were 100 to 150‐nm‐thick films processed by spin coating 16 mg mL^−1^ chloroform (CF) solutions at 3000 rpm (thickness measurements for an as‐cast sample is included in the Figure S1, Supporting Information). All samples analyzed were thermally annealed at 100 °C for 10 min in order to mimic the thermal protocols applied for the fabrication of OTFTs.

**Figure 1:**
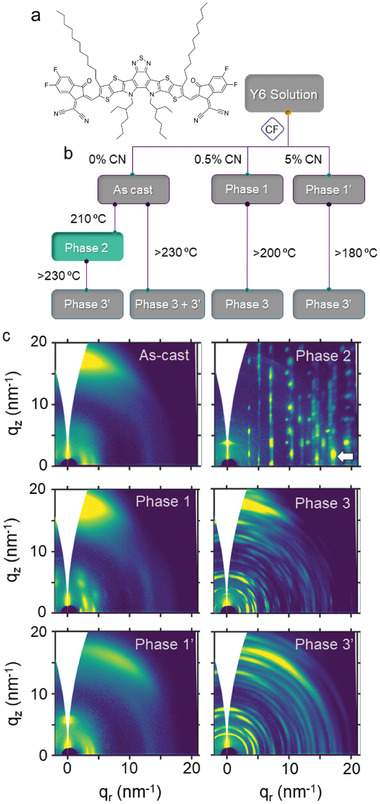
a) Chemical structure of Y6. b) Processing routes toward the different packing motifs/microstructures found. c) 2D‐GIWAXS patterns the packing motifs/microstructures found. The diffraction associated to main *π*–*π* stacking in phase 2 crystals is highlighted with an arrow.

Overall, we find six packing variations in Y6 films: five crystalline phases and a partially ordered microstructure denoted here as the “as‐cast” microstructure. The as‐cast microstructure and crystalline phases 1 and 1′ result via solution processing Y6 under different conditions, while phases 2, 3, and 3′ develop upon thermally annealing the above. Shown in **Figure** **1**b are suitable processing routes toward the formation of the microstructures/polymorphs found, based on the experimental data included in Figures S2–S7, Supporting Information. Crystalline phases 1 and 1′ develop upon casting Y6 solutions in CF comprising 0.5% and 5% of chloronaphtalene respectively. Likewise, we observed the development of phase 1 upon CF vapor annealing treatment of the as‐cast microstructure (data shown in Figure S8, Supporting Information). Phase 2 results from heating the as‐cast microstructure at temperatures ranging from 200 to 220 °C. Phases 3 and 3′, on the other hand, are obtained upon thermal treatment from the as‐cast microstructure and phase 2 at temperatures above 230 °C, and from phases 1 and 1′at temperatures above 200 and 180 °C respectively.

Structural differences between the various packing motifs/microstructures can be deduced from grazing Incidence Wide Angle X‐Ray Scattering (GIWAXS) patterns, included in Figure 1c, polarized optical microscopy (POM) and fast scanning calorimetry (FSC) (included in Figures S9 and S10, Supporting Information, respectively). The few and diffuse diffraction maxima of the as‐cast microstructure suggest that this is a solid structure with a low degree of structural order that is kinetically quenched during spin‐casting. While lacking long‐range order, Y6 molecules clearly exhibit a certain degree of preferential face‐on orientation. Moreover, FSC thermograms exhibit a signal at about 200 °C that can be associated with the glass transition (FSC data will be shown later in Figure 4), hence we argue that the as‐cast microstructure corresponds to a Y6 glassy phase exhibiting some degree of molecular order/orientation. Interestingly, the strong tendency of Y6 to form partially oriented molecular clusters has been recently suggested by molecular dynamics simulations.^[^ ^9^ ^]^ We must also note that this phase is frequently observed in binary blends in OSC devices.^[^ ^4^, ^6^, ^10^ ^]^

Similarly to other NFAs forming mesh‐like structures,^[^ ^3^, ^4^ ^]^ the GIWAXS patterns of crystalline phases produced during solution casting, that is, phase 1 and 1′, are characterized by multiple diffraction maxima in the low‐*q* region and a single, broad peak in the high‐*q* region, likely associated with the diffraction from *π*‐stacked planes. High‐temperature packing motifs, that is, phases 2, 3, and 3′, however, seem to have more symmetry elements according to their higher number of diffraction peaks. We must note that phase 3 is the polymorph with more similarity to the reported single‐crystal packings.^[^ ^4^, ^6^, ^7^, ^11^ ^]^ Hence, like many other organic semiconductors, Y6 exhibit a rich polymorphism. Typically, the presence of distinct polymorphs in organic semiconductors results from the fact that dominant interactions between these kinds of conjugated molecules are typically Van de Waals interactions and electrostatic interactions, which are weak and nondirectional. Consequently, molecules tend to have many options to combine each other, which results in different packing motifs with similar free energy levels.

**Figure 2:**
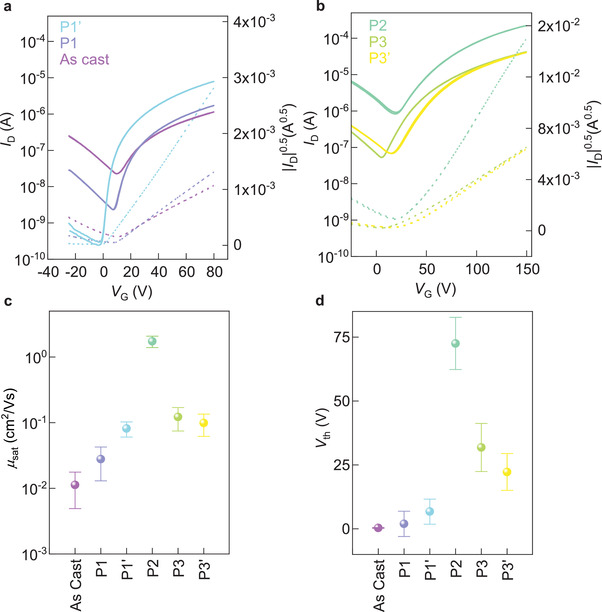
Top‐gate bottom‐contact Y6 OTFTs. Representative transfer characteristics and corresponding |*I*_D_|^0.5^ versus *V*_G_ plots measured for a) “as‐cast,” phase 1, phase 1′ measured at a drain voltage, *V*_D_, of 80 V and b) phase 2, phase 3, phase 3′ measured at *V*_D_ = 150 V. c) Corresponding charge‐carrier mobilities (*µs*_at_) and d) threshold voltage (*V*_th_) extracted in saturation regime, at *V*_G_ = 80 V and *V*_D_ = 80 V for “as‐cast”, phase 1, phase 1′ and at *V*_G_ = 150 V and *V*_D_ = 150 V for phase 2, phase 3, and phase 3′.

To investigate the charge transport properties of the different polymorphs/microstructures, we tested bottom‐contact, top‐gate Y6 OTFTs fabricated on glass with gold source and drain contacts and Cytop as the dielectric. **Figure** **2**a,b shows the transfer characteristics and corresponding plots of the square root of the channel current (|*I*_D_|^0.5^) versus gate voltage (*V*_G_) for the as‐cast microstructure and crystalline phases 1, 1′, 2, 3, and 3′. OTFTs transfer and output characteristics are, moreover, included in Figures S11 and S12, Supporting Information, respectively.

Several relevant information can be readily obtained from the data analysis. First, all polymorphs lead to devices exhibiting proper transistor operation and good *I*–*V* linearity. As shown in Figure 2c (and in Table S1, Supporting Information), in general, the *μ*_e_ is increased for high temperature polymorphs, that is, phases 2, 3, and 3′ (*μ*_e_ > 0.1 cm^2^ V^−1^ s^−1^), compared to the low temperature phases/microstructures 1, 1′and as‐cast (*μ*_e_ ≈ 0.01–0.1 cm^2^ V^−1^ s^−1^). This agrees with the standard understanding that transport properties of crystalline molecular semiconductors improve upon enhancement of the crystalline quality. Especially worth noting is that OTFTs comprising phase 2 exhibit a remarkably high *μ*_e_ = 1.73 ± 0.34, with best devices reaching 2.4 cm^2^ V^−1^ s^−1^. This value is comparable, and even higher than that reported for single crystal Y6 OFETs.^[^ ^7^ ^]^ Interestingly, phase 2 shows moreover a particularly pronounced ambipolarity, with hole mobility approaching 1 cm^2^ V^−1^ s^−1^, when extracted at negative gate bias and positive drain voltage (Figure S13, Supporting Information), owing to the accumulation of holes occurring as a consequence to a change of sign (from positive to negative) of the effective gate potential within the channel. The proper p‐type unipolar operation could not be measured, probably due to a large charge injection barrier of holes in the highest occupied molecular orbital, HOMO, of Y6. It is worth mentioning that a sizable energetic barrier occurs also with the lowest unoccupied molecular orbital, of this molecule, leading to a non‐negligible contact resistance, as it can be deducted from the S‐shape of the output characteristics (Figure S11, Supporting Information). As expected, the contact limitations become more severe for devices showing higher mobility values.^[^ ^12^ ^]^ Simultaneously, the threshold voltage (*V*_th_) progressively increases toward positive voltage for polymorphs exhibiting increased mobility (Figure 2c,d). We must note that the extraction of charge carrier mobility is not hindered by contact resistance in our devices, as the *I*_D_–*V*_G_ curves preserve good linearity without the formation of kinks or double slopes. Nevertheless, the slope of the current (hence the mobility) shows a moderate gate dependence, probably related to disorder and contact resistance.

In order to rationalize the outstanding electrical performance of phase 2, we analyzed in detail its structural and morphological characteristics and compared to those of the other microstructures. A quick look at the 2D GIWAXS patterns included in Figure 1c already suggests major structural differences. In contrast to the arc‐like diffraction maxima of the GIWAXS patterns for the as‐cast structure and the phases 1, 1′, 3, and 3′, the pattern for phase 2 exhibits discrete point‐like diffraction maxima arranged in columns, which clearly indicates a superior crystalline order, hence, likely reduced static disorder and lower trap density.

**Figure 3:**
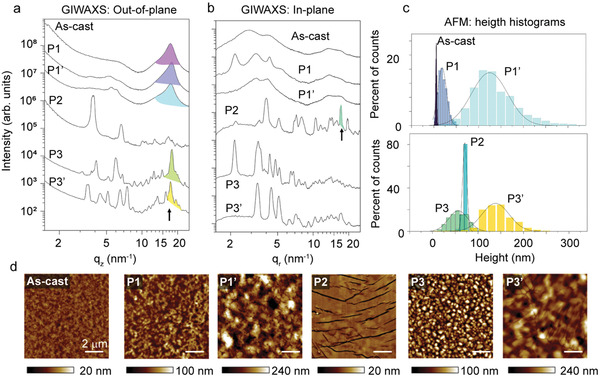
GIWAXS profiles along the a) out‐of‐plane and b) in‐plane directions for different packing motifs. Diffraction peaks associated with the *π*‐stacked planes are shadowed and highlighted with arrows. c) Height histograms obtained from the d) AFM data. Height distributions in (c) are fitted to Gaussian curves. All scale bars in (d) correspond to 2 µm.

Moreover, unlike the rest of the phases/microstructures, phase 2 films are oriented so that the overlap of *π*‐orbitals occurs along the plane of the film, favoring in‐plane charge transport and hence the percolation of charges in the channel of TFTs.^[^ ^13^ ^]^ We deduced this from the GIWAXS profiles along the out‐of‐plane and in‐plane directions. **Figure** **3**a shows that the *π*–*π* peaks for phases 1, 1′, 3, and 3′, and the “as‐cast” appear predominantly along the out‐of‐plane direction, hence, they exhibit negative Herrmann orientation parameters^[^ ^14^ ^]^ (**Table** **1**). Contrarily, the *π*–*π* peak of phase 2 is solely visible when the in‐plane scattering is analyzed, that is, in Figure 3b. We must note that we assumed the peak at *q* = 17.1 nm^−1^, indexed as (‐3‐12) according to our fitting (Figure S14, Supporting Information),^[^ ^15^ ^]^ as the peak associated with the *π*‐stacked planes in phase 2. The lattice parameters of the fitted unit cell are summarized in Table S2, Supporting Information.

**Table 1:** Structural and morphological parameters of Y6 packing motifs extracted from GIWAXS and AFM analysis

Phase	*q*_*π*–*π*_ [nm^−1^]	*d*_*π*–*π*_ [nm]	CCL_*π*–*π*_ [nm]	Herrmann parameter [*π*–*π*]	Roughness [nm]
As‐cast	17.4	0.36	2.2	−0.31	1.4
Phase 1	17.8	0.35	2.8	−0.35	9.7
Phase 1′	17.4	0.36	2. 1	−0.15	40.4
Phase 2	17.1	0.37	18.9	0.16	4.8
Phase 3	17.8	0.35	7.4	−0.34	21.6
Phase 3′	17.4	0.36	7.1	−0.16	30.0

Equally revealing is the comparison of the crystal coherence length of *π*‐stacked planes (CCL_*π*–*π*_) between the different phases, as determined with the Scherrer equation^[^ ^16^ ^]^ (Table 1). While CCL_*π*–*π*_ for phase 1 and 1′ films amount to 2–3 nm and those for phases 3 and 3′are about 7 nm, a noteworthy value of 18.9 nm is measured for phase 2. Clearly, the larger CCL_*π*–*π*_ values of crystals oriented with the *π*–*π* stack parallel to the substrate favors the charge transport along this direction.

The morphological analysis of the top surface of Y6 films, where the transport channel of our top‐gate OTFTs sets in, provides further insights into the superior performance of phase 2. Atomic force microscopy (AFM) height images, included in Figure 3d, show the markedly distinctive surface topography of phase 2 compared to the rest of the phases. Specifically, continuous domains over length scales that exceed tens of microns are observed in the former, whereas much smaller domains, with a higher amount of grain boundaries, are detected for the rest of polymorphs/microstructures. Moreover, the height histograms for phases 1, 1′, 3, and 3′ exhibit broad distribution peaks, denoting regions with large height variations, that is, large roughness, whereas histograms for phase 2 exhibit a very narrow distribution of heights, corresponding to large flat terrace‐like regions (Figure 3c,d and Table 1).

Hence, we argue that the molecularly flat, highly crystalline, and highly textured domains featuring sizes comparable to the channel length of our OTFTs devices are expected to play an important role in the enhancement in charge carrier mobility exhibited by phase 2 films.

Having established that the outstanding charge transport properties of phase 2 films are linked to superior structural properties, we set on to investigate the origin of this particular structure/morphology. Our data suggest that phase 2 develops from a partially ordered Y6 melt, which is formed when the “as‐cast” microstructure is heated at temperatures above its *T*_g_ (we recall that the as‐cast microstructure is a glassy phase and, as such, it undergoes glass transition). Hence, the already ordered/oriented molecules in the crystallizing melt likely guide the molecular order and orientation during the crystallization into phase 2, so that highly crystalline and textured films are created. Moreover, this templating effect may be further amplified by the low nucleation density of phase 2, which would enable the few propagating crystals to extend over large areas without interfering with further growing crystals.

**Figure 4:**
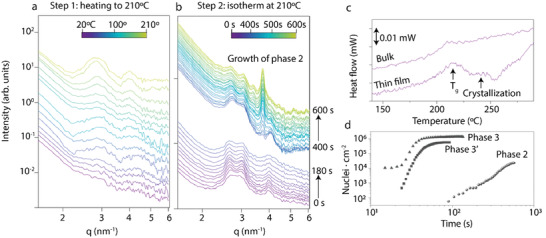
GIWAXS patterns (azimuthal integrations) recorded a) as heating the as‐cast microstructure from to 20 to 210 °C at 20 °C min^−1^, and b) during an isothermal step at 210 °C. c) FSC heating traces at 1000 °C s^−1^ for a bulk Y6 sample that was previously cooled down at 10 000 °C s^−1^ and for the as‐cast microstructure (this curve corresponds to the 1^st^ heating scan of a spin‐coated Y6 film). d) Evolution of the density of nuclei of 2, 3, and 3′ crystals isothermally formed at 210 °C (experimental data extracted from POM)

We base our interpretation that phase 2 originates from an oriented liquid on the experimental results shown in **Figure** **4**. We have already illustrated that phase 2 cannot develop via a polymorphic transition from phases 1 or 1′ (Figures S2–S7, Supporting Information). Instead, phase 2 solely results when the as‐cast microstructure is thermally treated. For that reason, we began our analysis by studying the effect of the temperature on the as‐cast microstructure. Figure 4a shows the in situ GIWAXS intensity (integrated along the azimuthal direction) collected during a heating step from 20 to 210 °C (at 20 °C min^−1^) (the 2D‐GIWAXS patterns are included in Figure S15, Supporting Information). It can be observed that the diffraction peaks between *q* = 2.5 and *q* = 4 nm^−1^, which originate from the molecular order/orientation in the as‐cast microstructure, increase in intensity and become narrower at 190–200 °C, that is, at temperatures slightly below those at which phase 2 can develop. We note that, the peaks above show up along the in‐plane direction and thus, they appear at *q*_r_ = 2.5 and *q*_r_ = 4 nm^−1^, respectively. This increase of the intensity and the narrowing of the peaks indicate that the order/orientation of the as‐cast material is increasing. Interestingly, this enhancement of order occurs at the same temperatures where FSC traces display the calorimetric signals from the devitrification (i.e., the glass transition) of Y6 during heating (Figure 4c). Hence, our in situ heating GIWAXS results are compatible with the as‐cast microstructure undergoing devitrification into an ordered/oriented melt in this temperature range. Moreover, this experiment (i.e., Figure 4a and Figure S15, Supporting Information) proves that Y6 film do not transform into phase 2 during heating to 210 °C, but a subsequent isothermal step at 210 °C is required.

The GIWAXS data collected during a subsequent isothermal step at 210 °C are shown in Figure 4b (2D‐GIWAXS patterns are included in Figure S16, Supporting Information). This set of data reveals that the formation of phase 2 crystals occurs in two steps: in the first step (initial ≈400 s), the order/orientation of the Y6 melt increases further. In the second step, diffraction peaks from phase 2 crystals begin to be visible; specifically, the (010) peak at *q* = 3.8 nm^−1^ (oriented at ≈30° from the equator as shown in Figure S16, Supporting Information). During the second step, the (010) peak becomes progressively more intense while the peaks from the ordered/oriented melt (between *q* = 2.5 and *q* = 4 nm^−1^, both oriented along the in‐plane direction) decrease concomitantly; suggesting, thus, that phase 2 develops directly from the ordered/oriented melt. This feature is fulfilled when solution casting conditions are chosen so that the film formation progresses quickly, for example, spin rates of 3000 and 5000 rpm. Conversely, a slower evaporation of the solvent, for example, via drop‐casting or spin coating at 500 rpm, leads to the formation of less oriented as‐cast microstructures that yield to phase 3, rather than phase 2, upon annealing at 210 °C (Figure S17, Supporting Information). Phase 3 obtained following this processing route exhibits the same charge carrier mobility produced upon annealing phase 1. A summary of the mobilities obtained from devices processed at different spinning rates is reported in Figure S18b, Supporting Information.

In a set of further experiments, we compare the crystallization kinetics of phases 2, 3, and 3′ at 210 °C. Because the birefringence of the low temperature microstructures/phases (i.e., as‐cast, 1 and 1′) is low, the development of the birefringent crystalline phases 2, 3, and 3′ can be readily monitored by POM (images are included in Figures S19–S21, Supporting Information, whereas the analysis is summarized in Figure 4d and **Table** **2**). Our data indicate that phase 2 crystals appear just after a long induction time, confirming the two‐step crystallization mechanism deduced from GIWAXS. Thus, the time required to reach 50% of the total crystalline conversion, *t*_0.5_, for phase 2 is one order of magnitude larger than those measured for phases 3 and 3′ at the same temperature. Furthermore, our data also indicates that phase 2 features a significantly lower maximum nucleation rate than phase 3 and 3′ (measured from the slopes of curves included in Figure 4d). Therefore, the nucleation density value is one order of magnitude smaller for phase 2 compared to the rest of the phases. As mentioned above, such a low density of nuclei in phase 2 films can explain why much larger crystals are found in these films.

**Table 2:** Crystallization parameters of crystalline phases 2, 3, and 3′ at 210 °C extracted from POM analysis

Phase	*t*_1/2_ [s]	Maximum nucleation rate [s^−1^]	Final density of nuclei [cm^−2^]	Phase
2	420	4	2.1 × 10^4^	2
3	37	169	1.1 × 10^6^	3
3′	47	43	5.1 × 10^5^	3′

Last, we would like to highlight that the outstanding structure and morphology of phase 2 films seems to be stable over long periods at room temperature. Indeed, a film stored for more than 10 months (in ambient conditions) still was solely comprised of phase 2 crystals (results included in the Figure S22, Supporting Information).

## 3 Conclusions

Solution‐processed OTFTs of the benchmark NFA used in OSCs, Y6, can exhibit a *μ*_e_ as high as 2.4 cm^2^ V^−1^ s^−1^ when it is crystallized in a readily achievable polymorph. This *μ*_e_ value is comparable to those of state‐of‐the‐art n‐channel OTFTs,^[^ ^8^ ^]^ hence opening up a plethora of new possibilities in the field of organic electronics for this well‐known material. Given the obvious need to find high *μ*_e_ organic materials^[^ ^8e^ ^]^—as presently champion *μ*_e_ values are still significantly lower than the counterpart hole mobility values—our work moreover introduces a promising approach to find materials fulfilling this condition: to research the solid‐state phase behavior of available NFAs and seek for polymorphs with high structural and morphological quality. In the context of these and previous results on small molecules and polymers,^[^ ^17^ ^]^ we argue that common experimental signatures of such high‐mobility phases may be the existence of molecularly flat, highly ordered, and textured domains featuring sizes comparable to those of TFT channels. These high‐mobility phases seem to stem from mesophases or ordered/oriented melts,^[^ ^18^ ^]^ in which the partially ordered molecules template the crystallization of a polymorph that features a low nucleation rate. Obviously, future studies will need to prove whether similar phases exist in other NFAs. Nonetheless, our work demonstrates that the NFAs commonly employed in OSCs are intrinsically high mobility materials and furthermore highlights their great potential not only in the photovoltaic arena but also in applications that require high *μ*_e_ values.

## 4 Experimental Section

### Materials

The NFA Y6 was supplied by Ossila Ltd., UK, and used as received. CF and 1‐chloronaphtalene were purchased from Merck and used as received. Cytop was purchased from AGC Chemicals, UK, and used as received.

### Sample Preparation

Y6 as received was dissolved in CF or a mixture of CF and chloronaphtalene (0.5% or 5% in volume) at a concentration of 16 mg mL^−1^. All the solutions were stirred at 45 °C for 1 h. Depending on the technique, silicon wafers or glass slides were used as a substrate. Both types of substrates were previously cleaned in an ultrasonic bath, submerged in acetone for 15 min, then in isopropyl alcohol for 15 min, and dried with compressed air. Thin films of Y6 were prepared by spin‐coating or drop‐casting. Spin‐coatings speeds were varied, from 500 to 5000 rpm, for 1 min. Then, unless it was specifically referred, thermal annealing procedures were applied immediately after deposition by placing the sample on a previously heated hot stage (Linkam Scientific Instruments Ltd.) at the target temperature.

### Organic Thin‐Film Transistors

Y6 was weighted in air, then the vial containing the material was annealed at ≈100 °C in a nitrogen‐filled glovebox for 30 min in an attempt to minimize the moisture absorbed by the material. Further, the Y6 solution was prepared in CF at a concentration of 16 mg mL^−1^ and stirred at 45 °C for 1 h. OTFTs were fabricated with a top‐gate, bottom‐contact architecture onto 2.54 × 2.54 cm^2^ glass substrates. The source and drain electrodes of Al/Au (5/35 nm) were deposited via thermal evaporation and the pattern was defined with shadow masks and cleaned by sonication in acetone and isopropanol. Y6 was then spin‐coated onto the substrates at 3000 rpm for 50 s if not specifically mentioned, before an annealing at 100 °C for 1 h and further thermal treatments to obtain the different polymorphs. Cytop was then spin‐coated at 2000 rpm for 60 s (≈900 nm) and annealed at 80 °C for 1 h. Finally, the gate electrode (Al) was thermally evaporated through a shadow mask to complete the device architecture and transferred to the measurement glove box with a transfer tube. Electrical characterization was conducted in a dry nitrogen‐filled glovebox using an Agilent B2902A semiconductor parameter analyzer. Devices were never exposed to air.

### Grazing Incidence Wide Angle X‐Ray Scattering

Diffraction patterns at grazing incidence were taken at the BL11, NCD‐SWEET, beamline at ALBA Synchrotron, Cerdanyola del Vallès (Spain). The energy beam was set at 12.4 keV (*λ* = 0.1nm) using a channel‐cut Si (1 1 1) monochromator. The incident angle was set at 0.12° to maximize the scattered signal from the organic material. GIWAXS patterns were taken with a Rayonix LX255‐HS area detector (pixel size of 88 microns), placed at 210 mm from the sample position. Exposition times were varied from 1 to 10 s, depending on the scattering intensity.

Prior to the GIWAXS analysis, the authors evaluated the penetration (in fact, the attenuation) of the X‐ray beam (considering the X‐ray refractive index of the materials and the energy of the beam) employing a software from Lawrence Berkeley National Laboratory's (https://henke.lbl.gov/optical_constants/atten2.html). The outcome is summarized in Figure S23, Supporting Information: The attenuation length, that is, the depth at which the beam intensity was dropped by a factor of 1/e, increased up to 1 *μ*m for incident angles >0.11°. This means that using an incident angle of 0.12°, like in the authors′ experiments, the X‐ray beam could penetrate and probe the whole volume of the film.

Hermans orientation parameters (HP) were calculated with azimuthal integrations of the *π*–*π* stacking reflection using Equation (1), where HP = −0.5 means orientation perpendicular to the substrate plane, HP = 0 isotropic, and HP = 1 a complete orientation parallel to the substrate plane.

(1) $$\mathrm{HP}=1-\frac{3}{2}\langle \mathrm{si}n^{2}\beta \rangle \langle \mathrm{si}n^{2}\beta \rangle =\frac{\int_{0}^{\pi /2}I\left(\beta \right)\mathrm{si}n^{3}\beta \sin\beta \;d\beta }{\int_{0}^{\pi /2}I\left(\beta \right)\beta }$$

### Polarized Light Optical Microscopy

The crystallization and morphology of samples were evaluated by POM (Zeiss Axio Scope A1). Samples were spin‐cast on glass from a 16 mg mL^−1^ solution at 3000 rpm. The glass slide was placed within a hot stage (Linkam Scientific Instruments Ltd.), which was positioned between the polarizer and the orthogonally oriented analyzer. For data analysis of POM images, the authors used a self‐written macro for ImageJ software. A proper color threshold was set on every image so the birefringent domains were isolated from the background and then, the size and number of birefringent particles were counted by using the particle analysis plugin, establishing a minimum size of 50 pixels for each particle.

### Atomic Force Microscopy

An AFM Dimension ICON with a Nanoscope V controller (Bruker) was used. Images were taken in Peak‐Force Tapping mode using ScanAsyst‐Air tips by Bruker (nominal tip radius = 2 nm, nominal frequency = 70 kHz, nominal spring constant = 0.4 N m^−1^).

### Fast Scanning Calorimetry

A Flash‐DSC 1 (Mettler Toledo) was used. Y6 was deposited in the form of thin‐film or in bulk on MultiSTAR UFS1 MEMS chip sensors. The experiments were performed under a 20 mL min^−1^ N_2_ gas flow. Details of each experiment are described in its corresponding section in the Supporting Information

## Conflict of Interest

The authors declare no conflict of interest.

## Supporting information

Supporting Information

## Data Availability

Research data are not shared.
