# Supplementary material for: Y6 Organic Thin‐Film Transistors with Electron Mobilities of 2.4 cm2 V−1 s−1 via Microstructural Tuning
Source: Adv Sci (Weinh). 2021 Dec 2;9(1):2104977. doi: 10.1002/advs.202104977 (PMC8728851; doi:10.1002/advs.202104977)
Supplement: Supplementary file 1 — Supporting Information [file ADVS-9-2104977-s001.pdf]

ADVANCED SCIENCE
Open Access

Supporting Information

for *Adv. Sci.*, DOI: 10.1002/advs.202104977

Y6 Organic Thin-Film Transistors with Electron Mobilities of 2.4 $cm^2 V^{-1} s^{-1}$ via Microstructural Tuning

Edgar Gutierrez-Fernandez, Alberto D. Scaccabarozzi,* Aniruddha Basu, Eduardo Solano, Thomas D. Anthopoulos, and Jaime Martín**

Supporting Information for

Y6 organic thin-film transistors with electron mobilities of 2.4 cm2·V-1·s-1 via microstructural tuning

Edgar Gutierrez-Fernandez, Alberto D. Scaccabarozzi*, Aniruddha Basu, Eduardo Solano, Thomas D. Anthopoulos, Jaime Martín**

Dr. Edgar Gutierrez-Fernandez, Dr. Jaime Martín
POLYMAT, University of the Basque Country UPV/EHU Av. de Tolosa 72, 20018, San Sebastián, Spain
Email: edgar.gutierrez@ehu.eus, jaime.martin.perez@udc.es

Dr. Alberto D. Scaccabarozzi, Dr. Aniruddha Basu, Prof. Thomas D. Anthopoulos
King Abdullah University of Science and Technology (KAUST), KAUST Solar Center (KSC), Thuwal 23955, Saudi Arabia
Email: alberto.scaccabarozzi@kaust.edu.sa

Eduardo Solano
ALBA Synchrotron Light Source, NCD-SWEET Beamline, Cerdanyola del Vallès, 08290, Spain.

Dr. Jaime Martín
Ikerbasque Basque Foundation for Science, Bilbao 48013, Spain.
Universidade da Coruña, Grupo de Polímeros, Centro de Investigacións Tecnolóxicas (CIT), Esteiro, 15471 Ferrol, Spain.

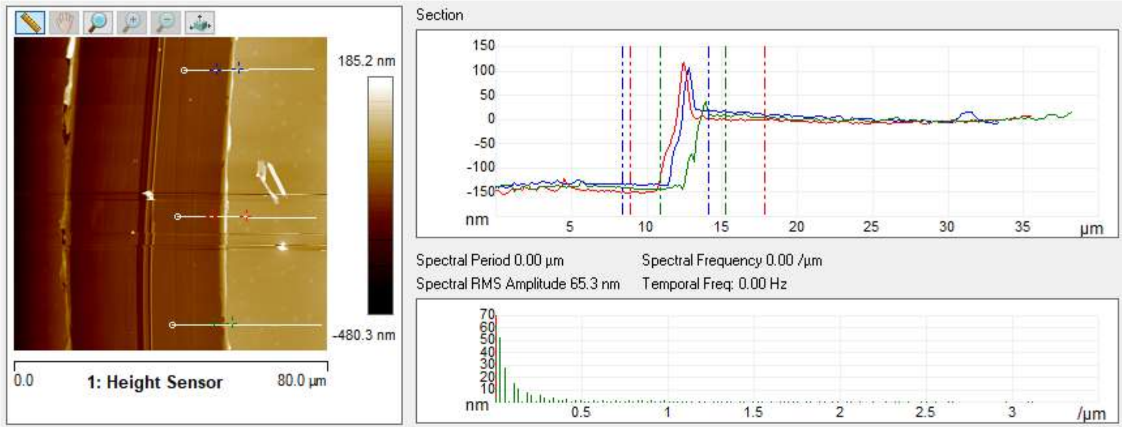

Figure S1 AFM scratch analysis of Y6 *as-cast* thin films. Film thickness was evaluated from height profiles.

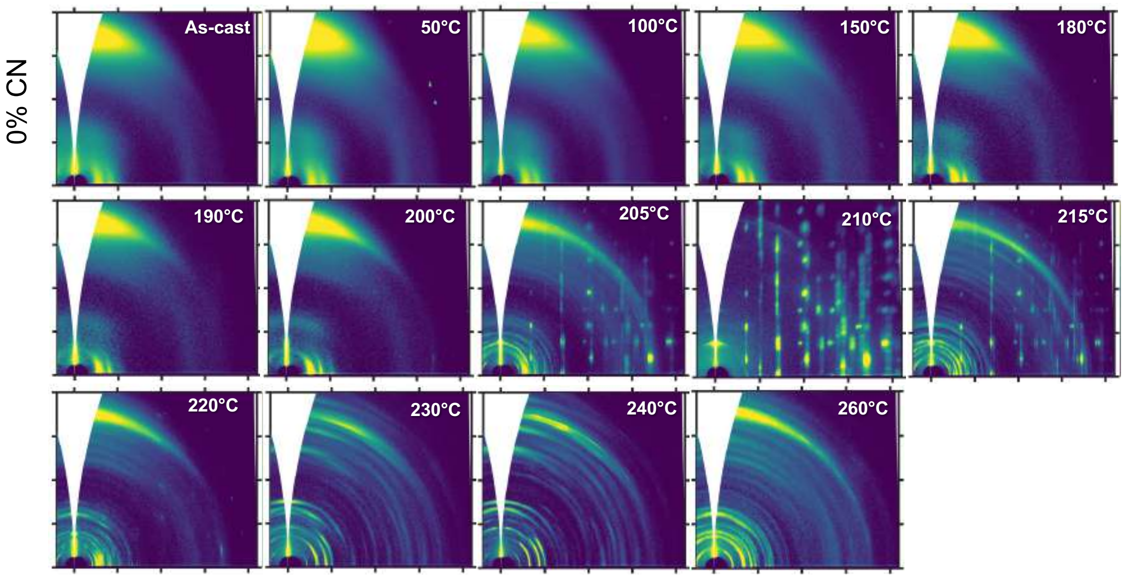

Figure S2. GIWAXS patterns of Y6 films (spin-cast from a 16 mg·mL^{-1} chloroform solution), acquired at room temperatures after being annealed at the indicated temperatures for 10 min.

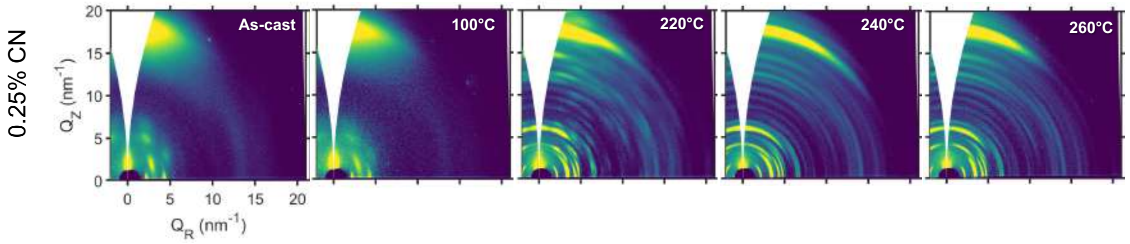

Figure S3. GIWAXS patterns of Y6 films spin-cast from a 16 mg·mL^{-1} chloroform solution containing 0.25 % of chloronaphtalene, acquired at room temperatures after being annealed at the indicated temperatures for 10 min.

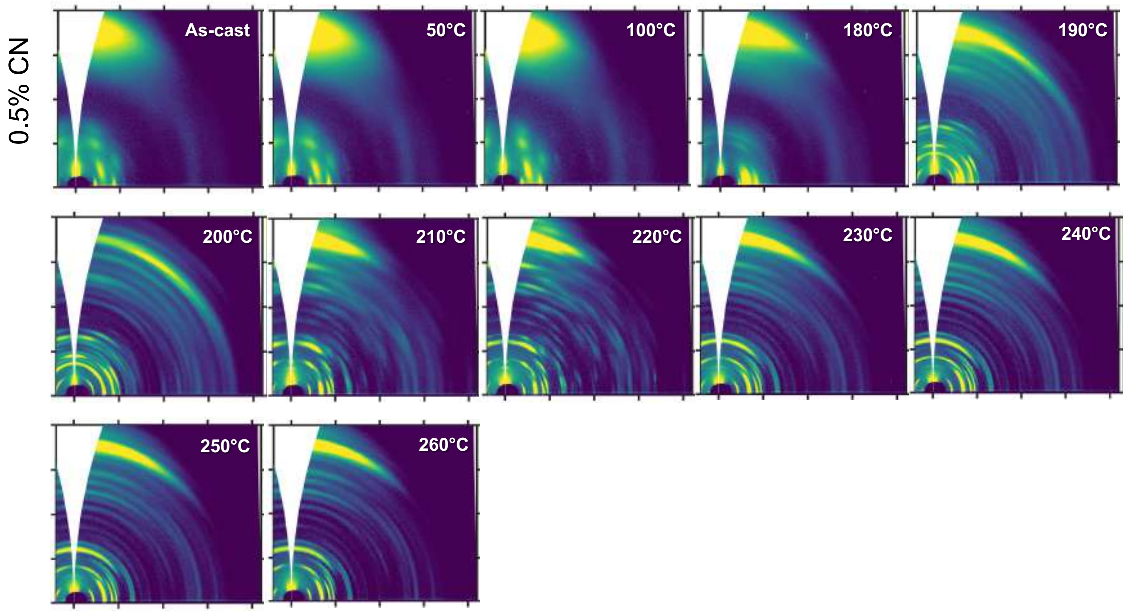

Figure S4. GIWAXS patterns of Y6 films spin-cast from a 16 mg·mL^{-1} chloroform solution containing 0.5 % of chloronaphtalene, acquired at room temperatures after being annealed at the indicated temperatures for 10 min.

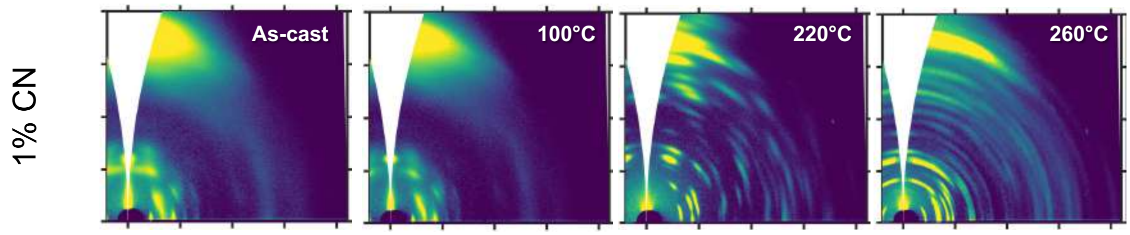

Figure S5. GIWAXS patterns of Y6 films spin-cast from a 16 mg·mL^{-1} chloroform solution containing 1 % of chloronaphtalene, acquired at room temperatures after being annealed at the indicated temperatures for 10 min.

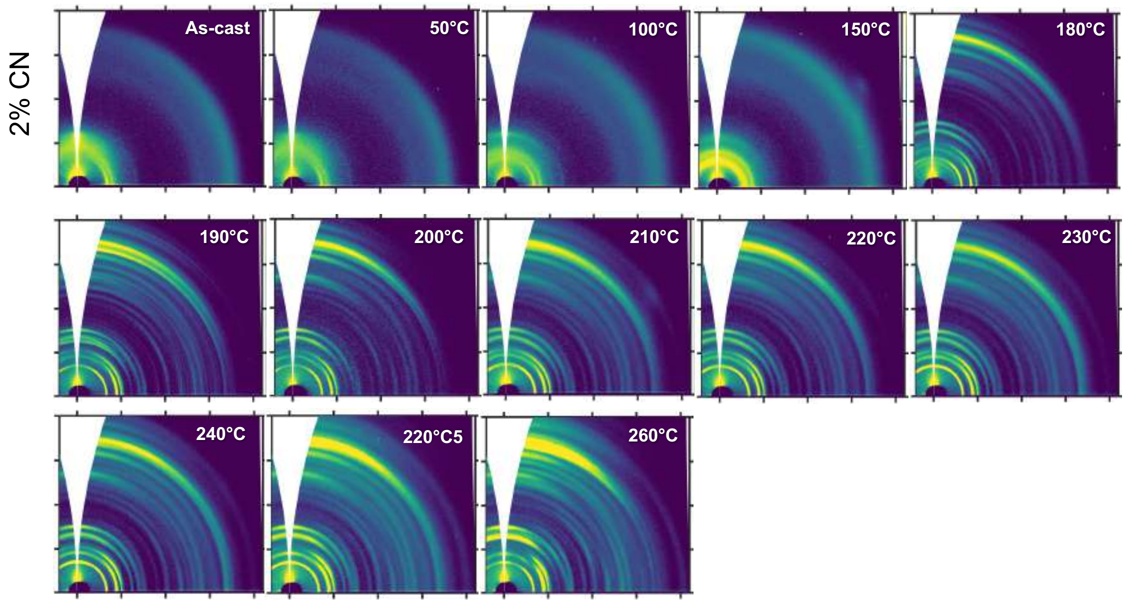

Figure S6. GIWAXS patterns of Y6 films spin-cast from a 16 mg·mL^{-1} chloroform solution containing 2% of chloronaphtalene, acquired at room temperatures after being annealed at the indicated temperatures for 10 min.

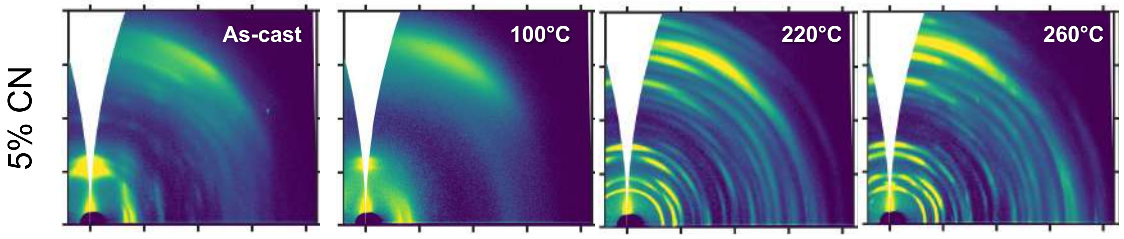

Figure S7. GIWAXS patterns of Y6 films spin-cast from a 16 mg·mL^{-1} chloroform solution containing 5 % of chloronaphtalene, acquired at room temperatures after being annealed at the indicated temperatures for 10 min.

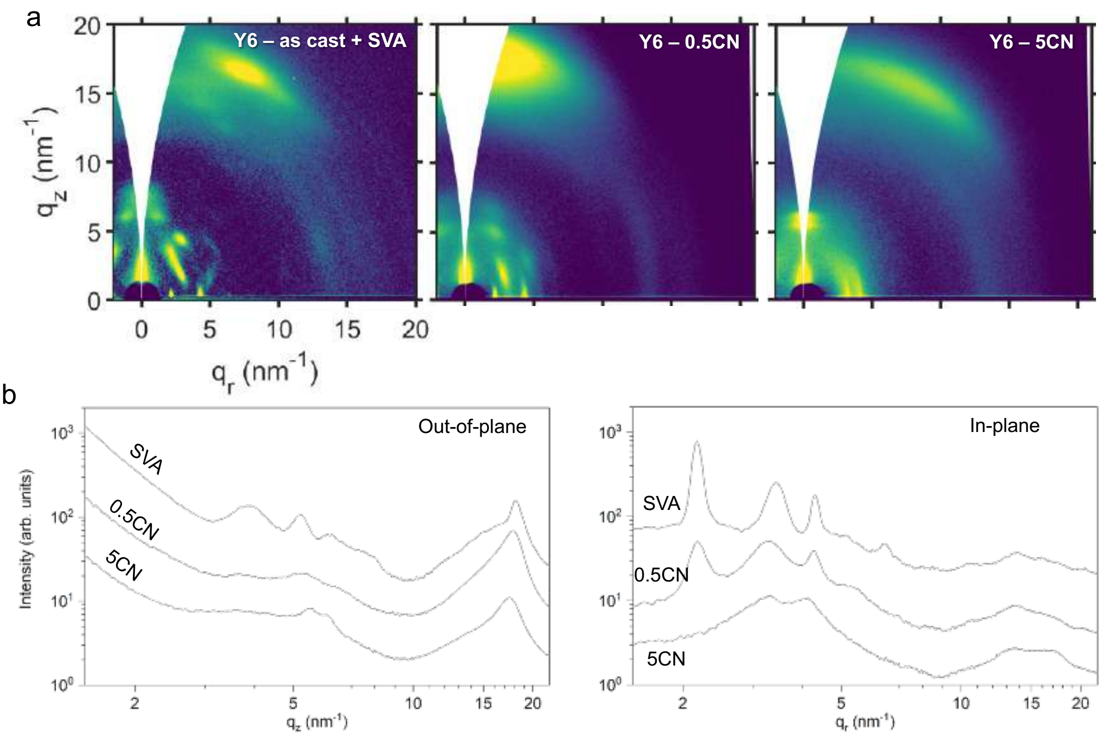

Figure S8. 2D GIWAXS pattern (a) and profiles (b) for Y6 as-cast microstructure treated with chloroform solvent annealing compared to phase 1 (0.5 CN) and phase 1´ (5CN).

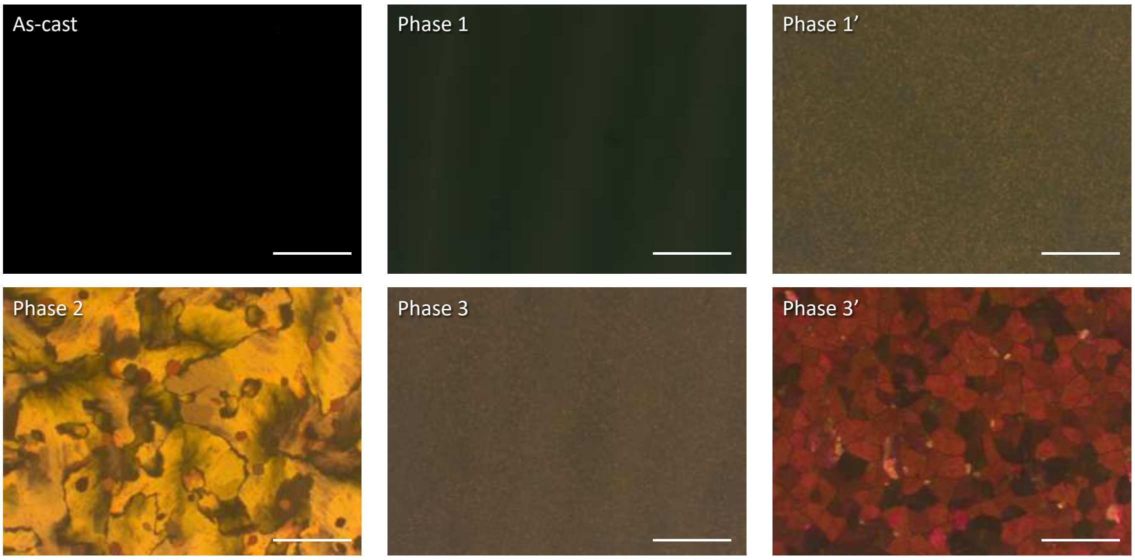

Figure S9. Polarized optical microscopy images of the phases/microstructures found in Y6. Scale bars correspond to 50 μm.

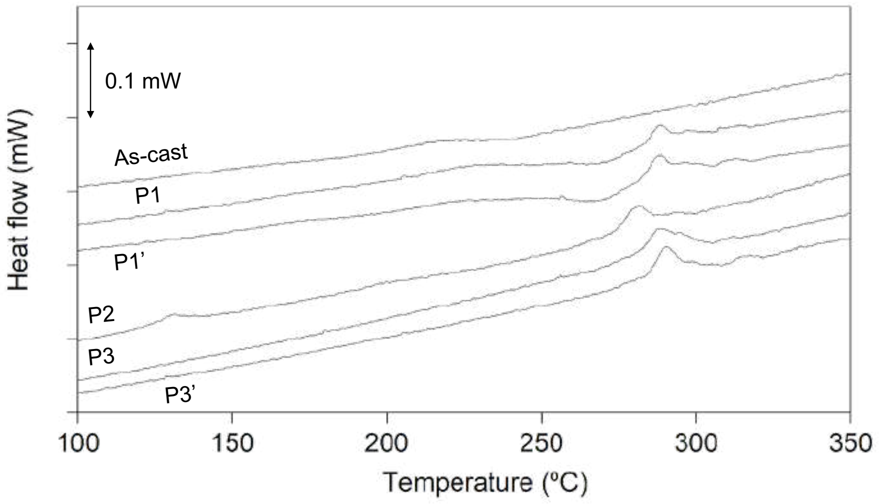

Figure S10. Fast scanning calorimetry: first heating curves for the various Y6 phases/microstructures.

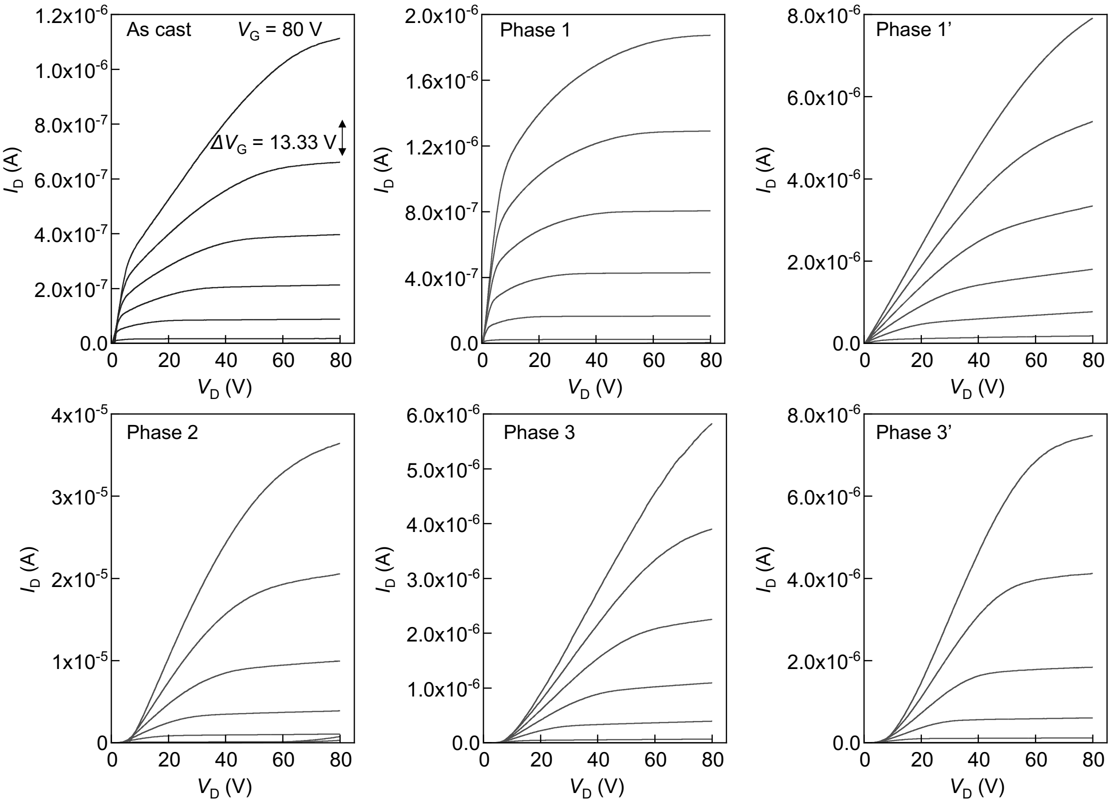

Figure S11. OTFTs output characteristics for different polymorphs/microstructures of Y6, as indicated in the top left corner of each panel. V_G is ranging from 0 V to 80 V, as indicated in top-left panel.

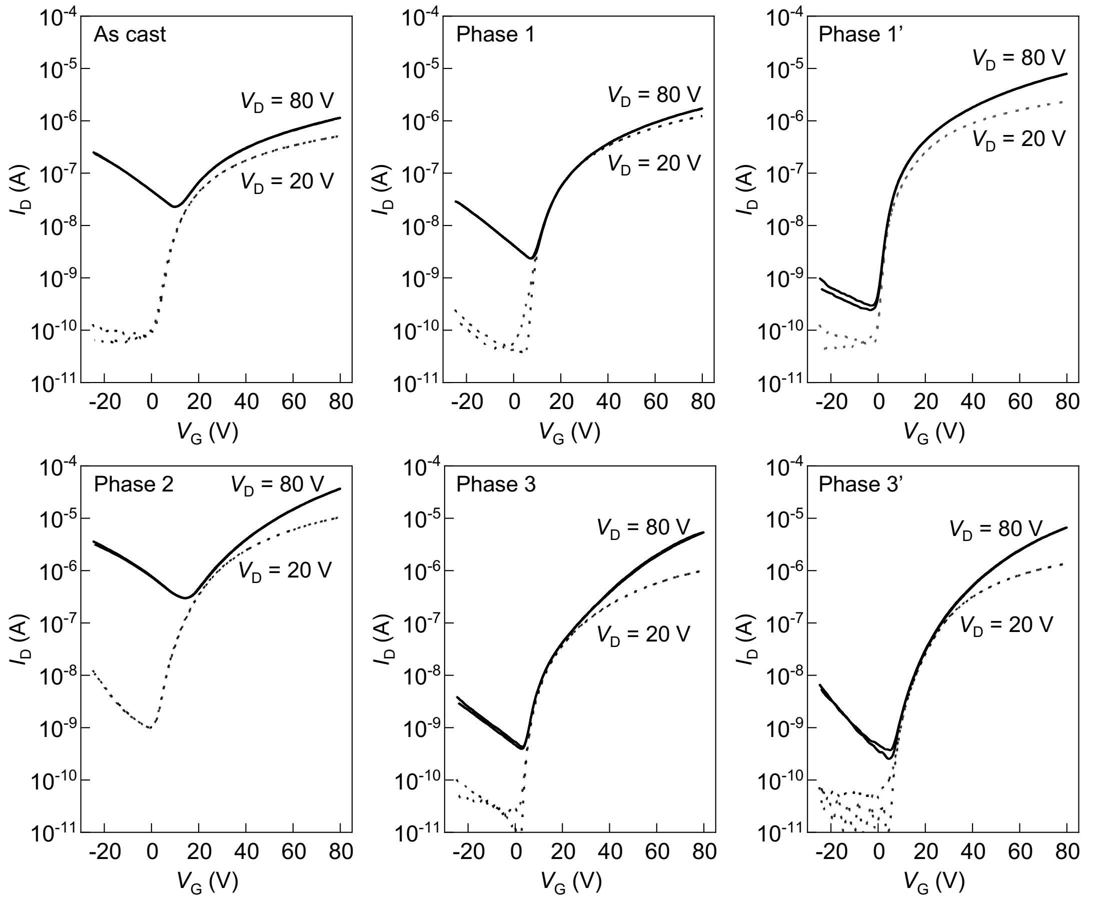

Figure S12: OTFTs transfer characteristics for different polymorphs/microstructures of Y6, as indicated in the top left corner of each panel. V_D is 20 V (dashed lines) and 80 V (solid lines).

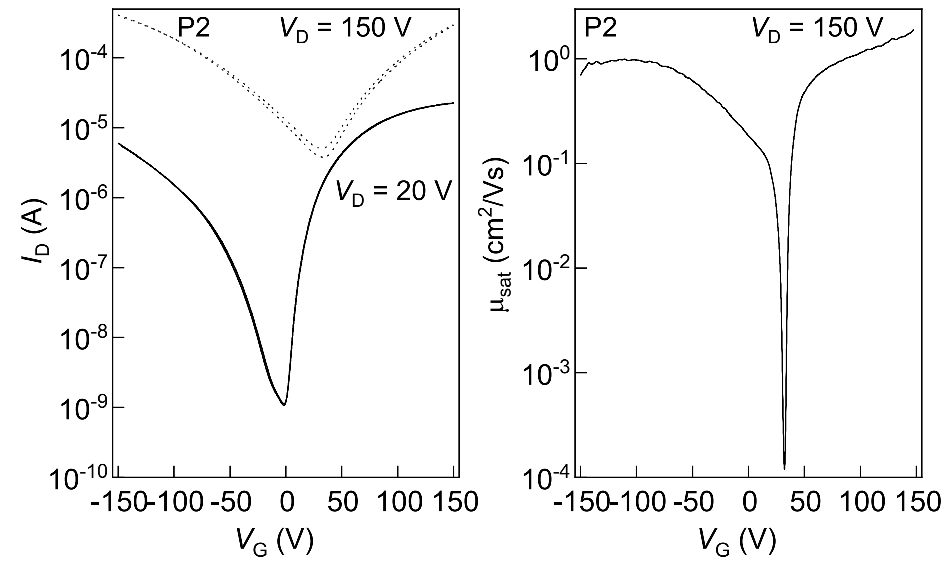

Figure S13: Left, OTFTs transfer characteristics of Y6 phase 2, measured at V_D = 20 V (solid line) and V_D = 150 V (dashed line) showing pronounced ambipolarity. Right, corresponding charge carrier mobility for V_D = 150 V.

Table S1. Charge-carrier mobilities (μ_{sat}) for “as cast”, phase 1, phase 1’ measured at V_D = 80 V and b) phase 2, phase 3, phase 3’ measured at V_D = 150 V.

	μ_{sat}	V_{th}
As cast	0.01 ± 0.006	0.35 ± 0.13
P1	0.03 ± 0.015	1.96 ± 4.96
P1’	0.08 ± 0.021	6.74 ± 4.89
P2	1.73 ± 0.343	72.49 ± 10.21
P3	0.12 ± 0.048	31.82 ± 9.42
P3’	0.09 ± 0.037	22.24 ± 7.21

Values of μ_{sat} and V_{th} are extracted at the maximum gate voltage.

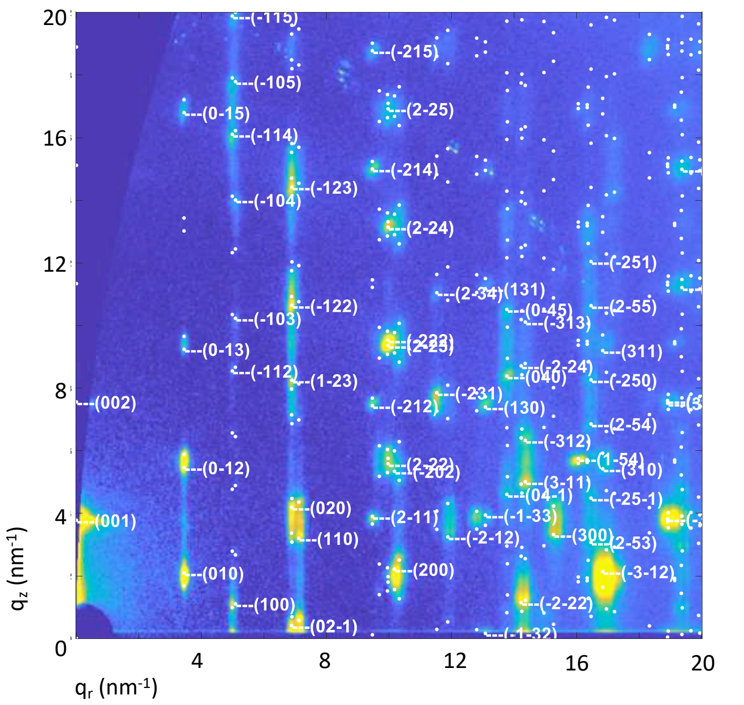

Figure S14. Fitted 2D GIWAXS patterns of Y6 phase 2

Table S2. Estimated lattice parameters of the fitted unit cell from the Y6 phase2.

	Y6 phase 2
a (nm)	1.33
b (nm)	1.96
c (nm)	1.95
alpha (°)	119.05
beta (°)	89.68
gamma (°)	111.57
volume (nm^3)	4.03

Unit cell sizes and lattice parameters for Y6 phase 2 were estimated using a MatLab script designed for this purpose (*Savikhin, V., et al., Journal of Applied Crystallography, 2020. 53: p. 1108)*.

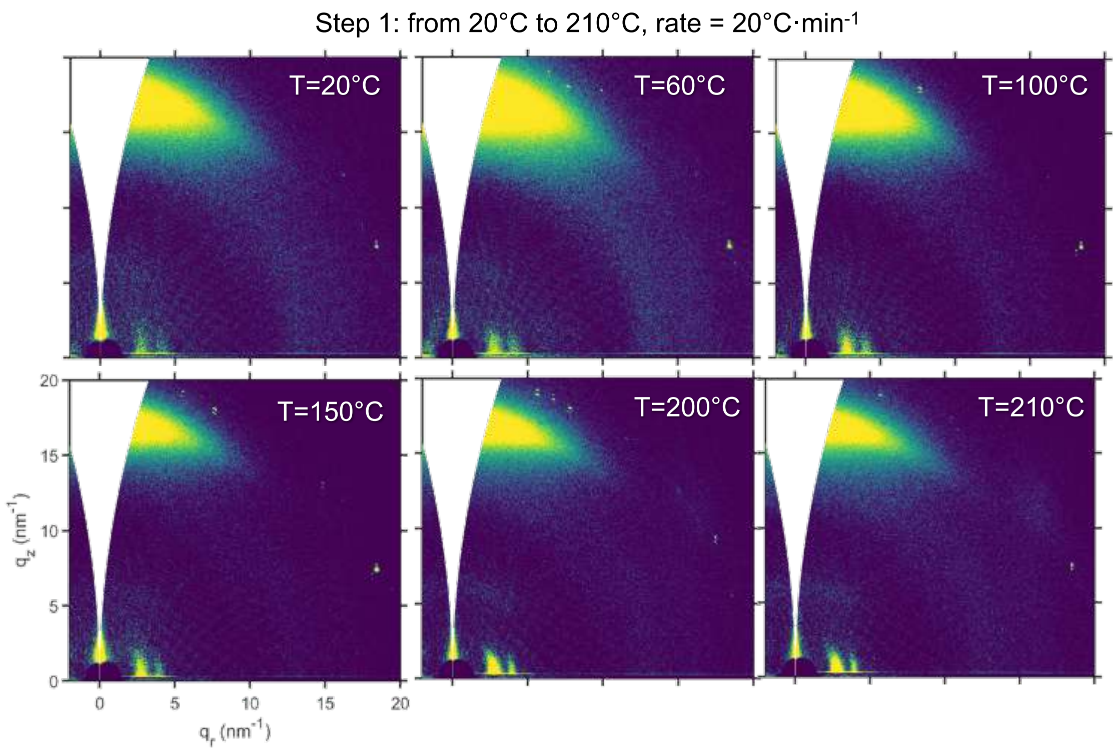

Figure S15. Formation of phase 2 from the as-cast sample (20°C), first step: heating ramp. *in situ* temperature-resolved GIWAXS data collected during a heating step from 20 ºC to 210 ºC (at 20 ºC/min)

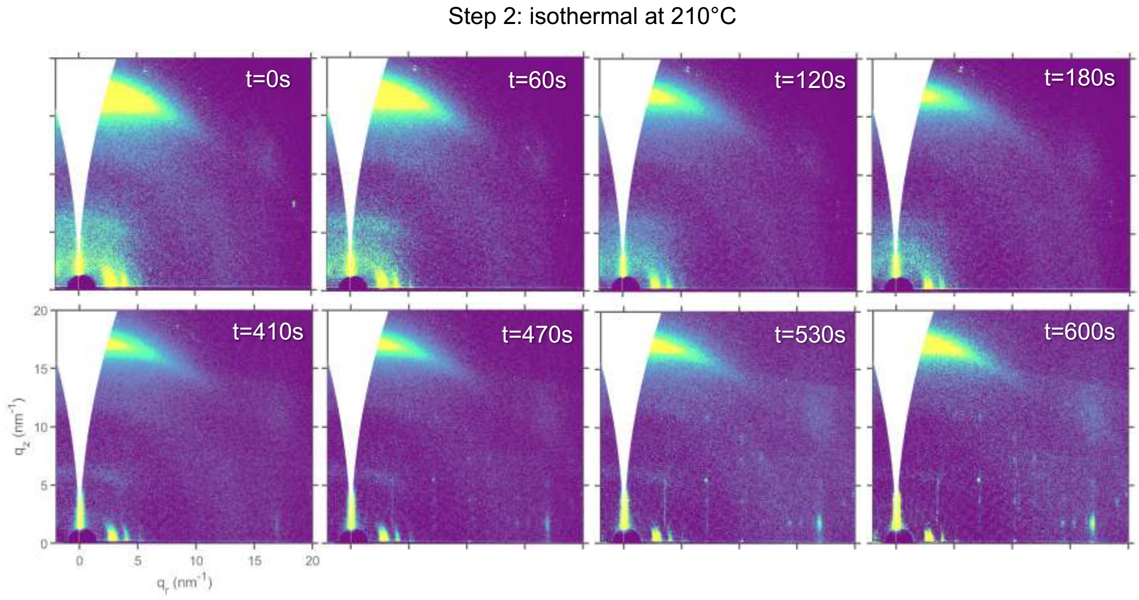

Figure S16. Formation of phase 2 from the as-cast sample (20°C), second step: isothermal at 210°C. GIWAXS data collected during an isothermal step at 210 ºC

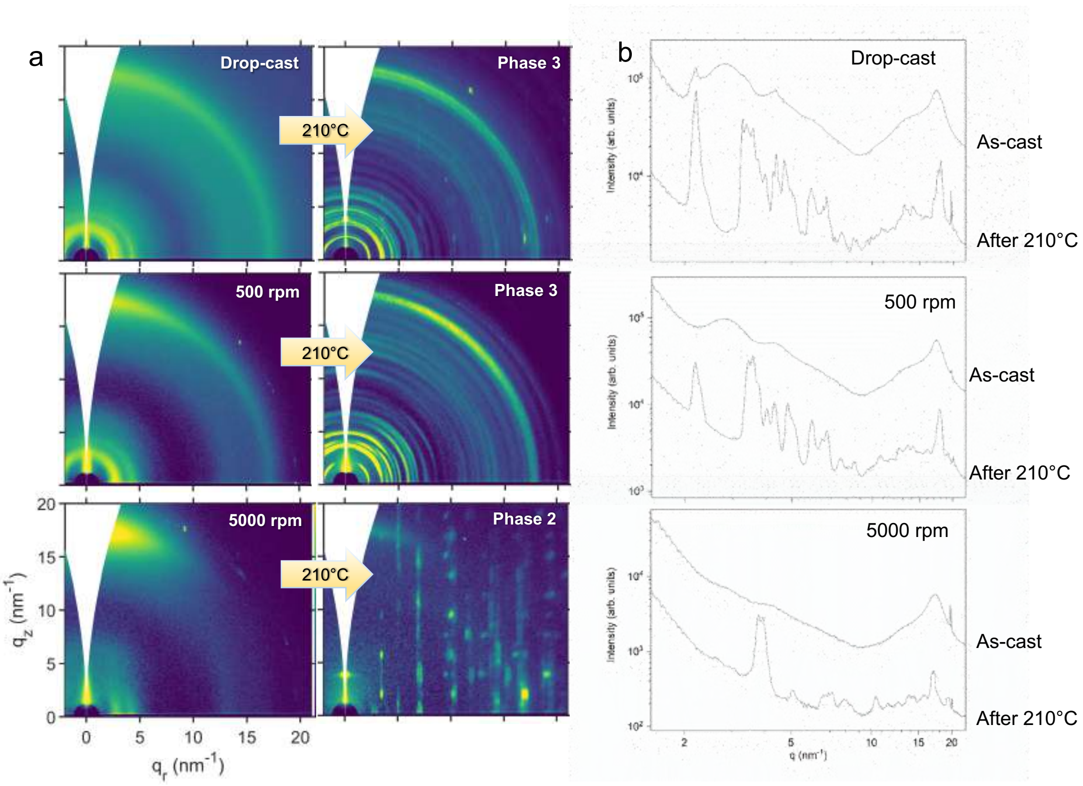

Figure S17. (a) GIWAXS patterns for films deposited by drop-cast, and spin coating at 500 and 5000 rpm as-cast (left panels) and annealed at 210 ºC (right panels). (b) Integrated GIWAXS profiles.

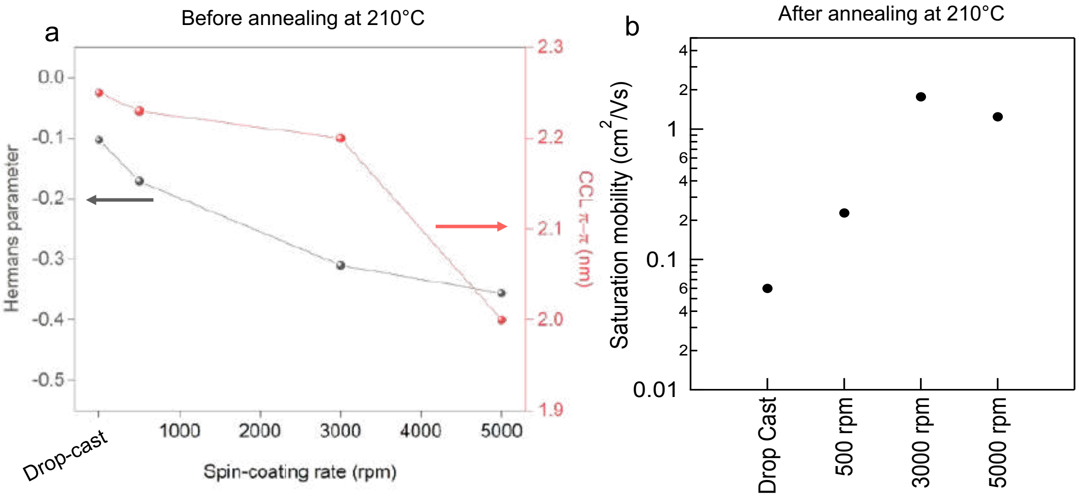

Figure S18. (a) Hermans parameter and CCL$_{\pi-\pi}$ vs spinning rate of samples cast at 0, 500, 5000 rpm (drop-cast is considered to be deposted at zero spinning rate). (b) Saturation mobility of the samples.

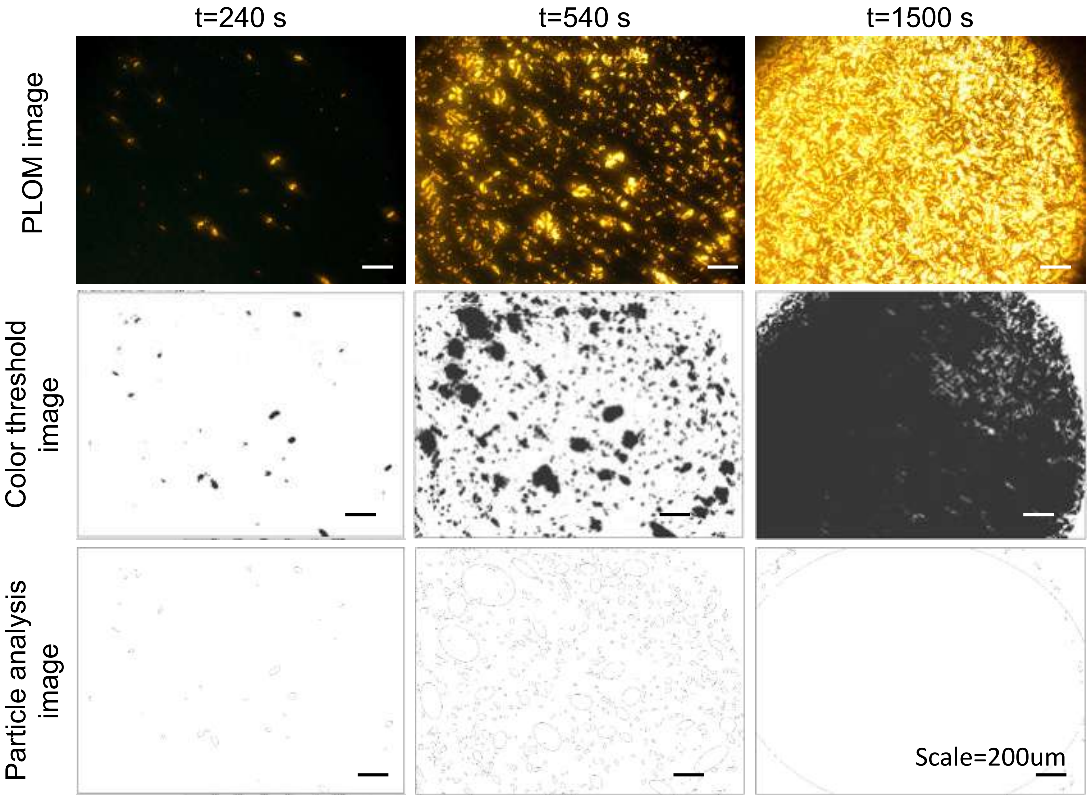

Figure S19. Polarized optical images during the isothermal crystallization of phase 2 at 210 ºC after 240, 540, and 1500 s (top panels). Central and bottom panels show different steps of the imagen analysis performed.

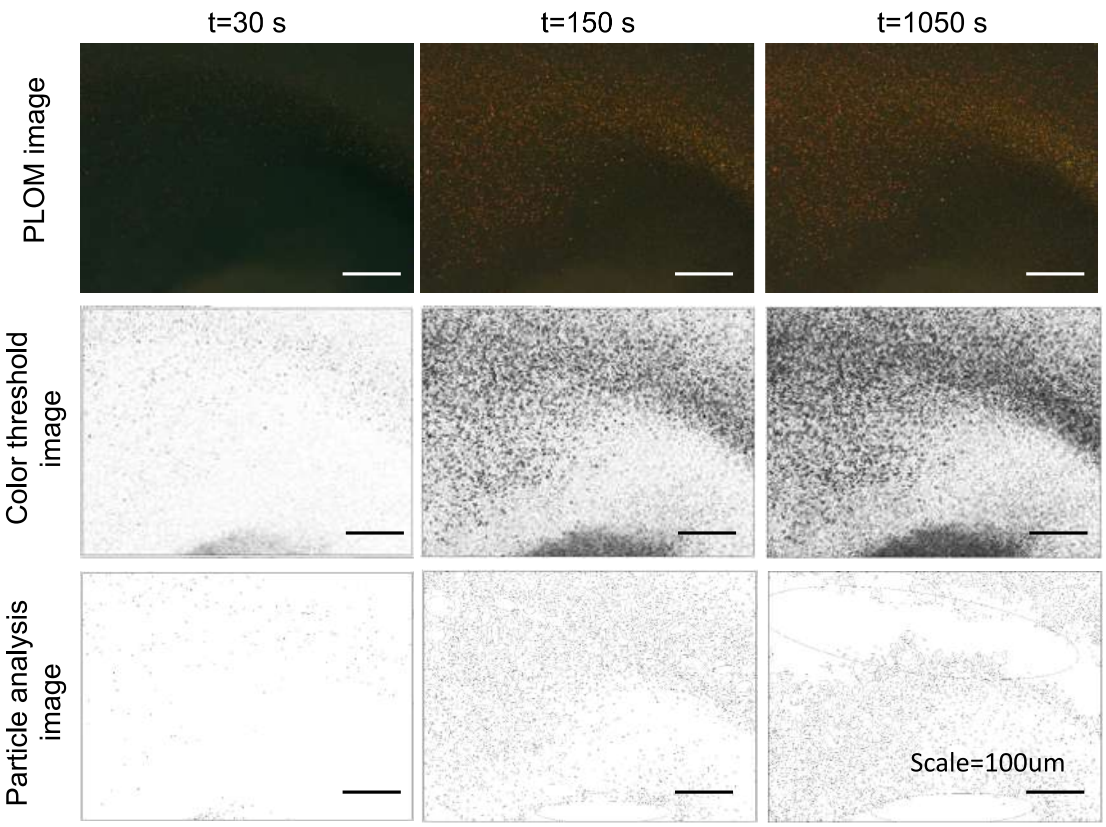

Figure S20. Polarized optical images during the isothermal crystallization of phase 3 at 210 ºC after 30, 150, and 1050 s (top panels). Central and bottom panels show different steps of the imagen analysis performed.

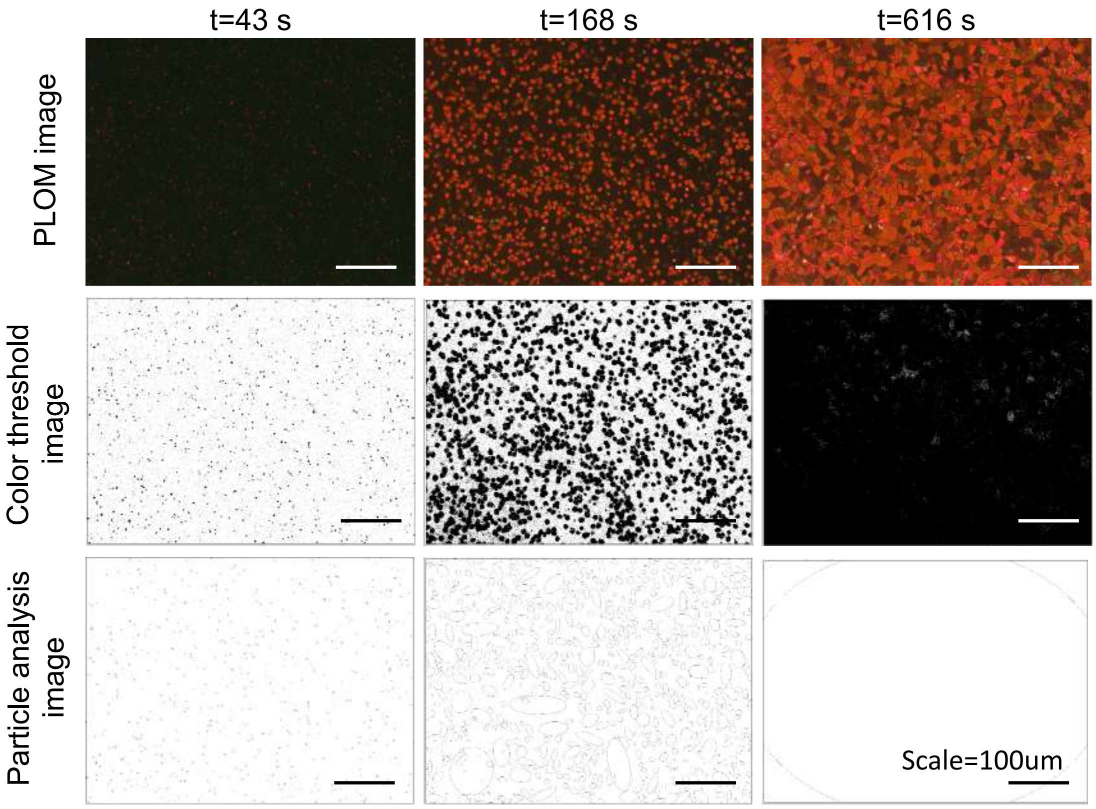

Figure S21. Polarized optical images during the isothermal crystallization of phase 3´ at 210 ºC after 43, 168, and 616 s (top panels). Central and bottom panels show different steps of the imagen analysis performed

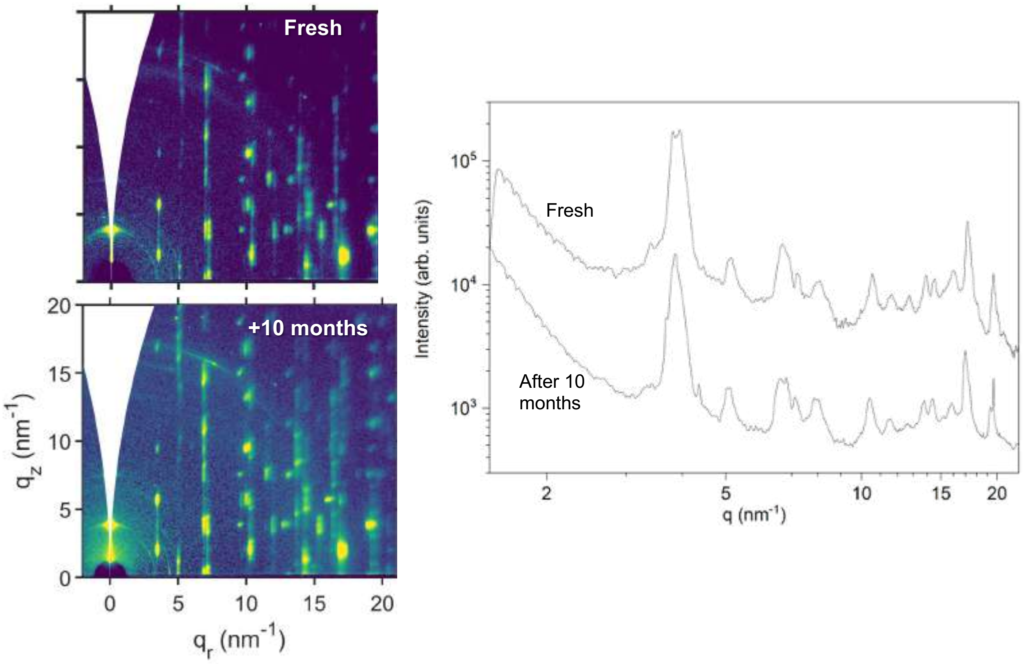

Figure S22. 2D GIWAXS patterns and integrated profiles for a phase 2 film freshly prepared and stored for 10 months at room temperature

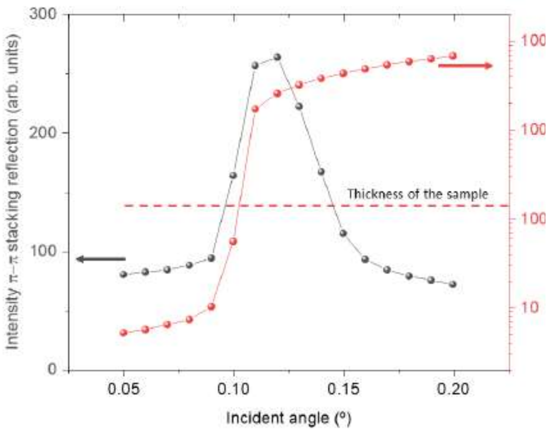

Figure 23 GIWAXS intensity of the π-π diffraction peak and attenuation length of the x-ray beam plotted vs the incident angle employed in the GIWAXS experiments**.** Source: https://henke.lbl.gov/optical_constants/atten2.html

Parameters used of the simulation:

Energy = 12.4 KeV (0.1 nm)
Chemical formula: C82H86F4N8O2S5 (BTP-4F, Y6)
Density = 1.1 g/cm3
